# Understanding the Molecular Progression of Chronic Traumatic Encephalopathy in Traumatic Brain Injury, Aging and Neurodegenerative Disease

**DOI:** 10.3390/ijms24031847

**Published:** 2023-01-17

**Authors:** FNU Ruchika, Siddharth Shah, Durga Neupane, Ruddra Vijay, Yusuf Mehkri, Brandon Lucke-Wold

**Affiliations:** Department of Neurosurgery, University of Florida, Gainesville, FL 32611, USA

**Keywords:** chronic traumatic encephalopathy, Tauopathy, molecular basis, neuroinflammation

## Abstract

Traumatic brain injury (TBI) is one of the leading causes of death and disability among children and adults in America. In addition, the acute morbidity caused by TBI is implicated in the development of devastating neuropsychiatric and neurodegenerative sequela. TBI is associated with the development of a neurodegenerative condition termed ‘Punch Drunk syndrome’ or ‘dementia pugilistica’, and the more recently renamed ‘chronic traumatic encephalopathy’. Chronic traumatic encephalopathy (CTE) is a slowly progressive neurodegenerative condition caused by a single or repetitive blow to the head. CTE was first described in boxers and was later found to be associated with other contact sports and military combat. It is defined by a constellation of symptoms consisting of mood disorders, cognitive impairment, and memory loss with or without sensorimotor changes. It is also a Tauopathy characterized by the deposition of hyperphosphorylated Tau protein in the form of neurofibrillary tangles, astrocytoma tangles, and abnormal neurites found in clusters around small vessels, typically at the sulcal depths. Oxidative stress, neuroinflammation, and glutaminergic toxicity caused due to the insult play a role in developing this pathology. Additionally, the changes in the brain due to aging also plays an important role in the development of this condition. In this review, we discuss the molecular mechanisms behind the development of CTE, as well as genetic and environmental influences on its pathophysiology.

## 1. Introduction

Chronic traumatic encephalopathy (CTE) is a progressive neurodegenerative condition caused by a single or multiple blows to the head. The pathophysiology of the development and progression of CTE is linked to the disruption of certain reparative molecular pathways. Traumatic brain injury (TBI) is the inciting factor leading to this disease, but there are other factors such as age that play a role in its progression. Oxidative injury plays a central role in brain damage after TBI, and the microglia play a central role in the reparative process. Disruption of this reparative ability of microglia and other neural pathways affects the ability of the brain to repair itself. Over time, repeated cycles of inflammation lead to pathological deposits called neurofibrillary tangles. This pathological finding is the basis of the diagnosis of CTE. In this review we explain the evolution of traumatic brain injury to CTE and the pathophysiology behind this evolution. We also highlight the important molecular mechanisms that play a vital role in the development of CTE along with a brief overview of aging and its effects on the progression of CTE. We also explore the relationship between CTE and other neurodegenerative conditions. The objective of this review is to provide a sound overview of our current understanding of the molecular progression of chronic traumatic encephalopathy in traumatic brain injury, aging, and neurodegenerative disease

## 2. Neuropathology of CTE

Chronic traumatic encephalopathy is diagnosed most accurately by a neuropathological examination of a brain tissue specimen [1]. The classical finding is an accumulation of hyperphosphorylated Tau protein in neurons and glial cells, mainly involving the perivascular areas and preferential deposition in the cortical sulci depths [2,3,4,5,6]. The specific isomer is cis p-Tau and was found to be produced after traumatic injury to the brain causing cell toxicity, synapse, and circuit dysfunction [6,7].

Studies of concussion and post-concussion syndrome in human subjects found that multifocal traumatic axonal injury is most commonly perivascular [8,9,10] with focal clusters of p-Tau in neurofibrillary tangles, pre-tangles, and neurites [11]. Progression is believed to begin with brain trauma, causing some Tau proteins to become dissociated from microtubules in axons via intracellular calcium influx, glutamate receptor-mediated mechanisms excitotoxicity, and kinase activation mediating hyperphosphorylation of intracellular Tau [12]. In addition, the Tau protein, which dissociates from microtubules, may undergo phosphorylation, misfolding, and aggregation and become proteolytically cleaved by calpains and caspases associated with neurotoxicity [13]. Furthermore, astrocytic p-Tau has been associated with age and not years of exposure to repetitive head impacts (RHI). Overall, this work supports the 2021 consensus requirement for neuronal p-Tau in the pathognomonic lesion of CTE.

**Figure 1:**
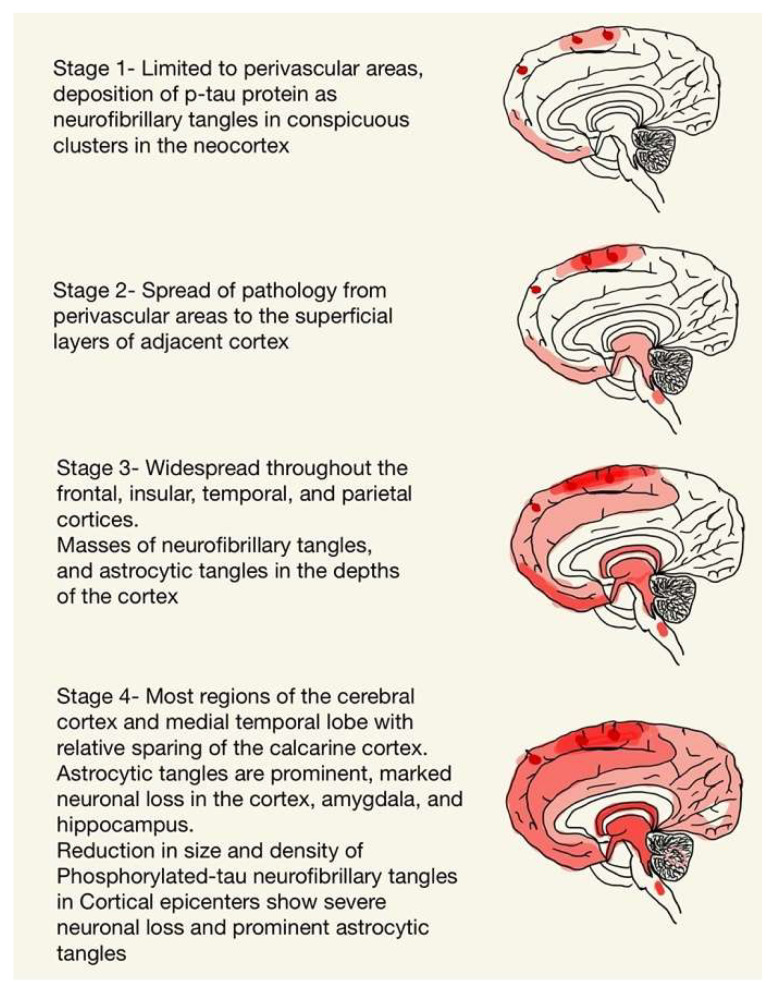
The progression of hyperphosphorylated p-Tau.

The production of cis p-Tau is believed to be initiated by ischemia and in regions that experience mechanical strain forces [14]. Ischemia causing oxidative stress might precede Tau deposition, and the lymphatic system might be a channel for the accumulation of p-Tau in the depths of sulci in a perivascular distribution [14]. The progression of hyperphosphorylated p-Tau can be divided into four phases, as depicted in Figure 1. The importance of isolated subpial p-Tau astrocytes in the depths of sulci without any neurofibrillary tangles near perivascular areas in the underlying cortex is unknown, but subpial p-Tau astrocytes at the deep cortical sulci is not a phenomenon found in normal aging and has been found in the brains of individuals with a history of chronic repetitive brain trauma [2,11]. The isoform profile of Tau and its phosphorylation state in CTE is like that in Alzheimer’s disease [15], and the neuronal p-Tau pathology shows immunoreactivity to both three repeats (3R) and four repeats (4R) Tau [11,16]. The 4R isoform of Tau is mainly expressed in astrocytes in the subpial region of deep sulci [11,16]. However, the neuronal abnormalities in the hippocampus appear to be primarily 4R Tau in CTE [11]. Its distribution is depicted in Figure 1.

**Figure 2:**
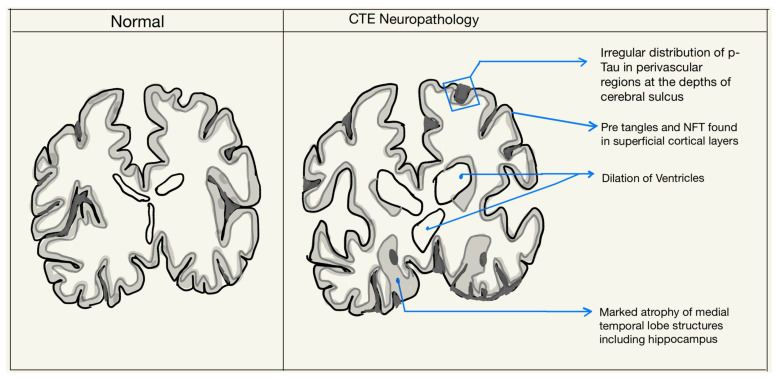
Neuropathological features in the diagnosis of CTE.

Grossly, the changes in the brain are not common in the early or mild stages of CTE. Lesions may be present in perivascular spaces in the white matter, mainly in the temporal lobe. Some macroscopic changes include reduction in brain weight, gray and white matter atrophy—typically severe in the frontal and anterior temporal lobes, as well as enlargement of the lateral and third ventricles, cavum septum pellucidum, and septal fenestrations. Other features seen are the atrophy of the thalamus, hypothalamus, and mammillary bodies, the thinning of the isthmus of the septum corpus callosum, and the depigmentation of the locus coeruleus and substantia nigra. Some of these changes are demonstrated in Figure 2 [2,11].

TDP 43 is TAR DNA-binding protein 43 and is also accumulated, causing TDP 43 immunoreactive inclusions [3,4]. It translocates from the nucleus to the cytoplasm, where it can become polyubiquitinated and hyperphosphorylated, resulting in the formation of pathological inclusion bodies [17]. These have been identified in patients with Alzheimer’s disease (AD), Parkinson’s disease (PD), amyotrophic lateral sclerosis (ALS), and chronic traumatic encephalopathy (CTE) patients [5,18].

Amyloid-β (Aβ) deposition has also been associated with CTE [1,3,5] with significant deposition documented in boxers [19] and American football players [20]. Although Aβ plaques have been identified in older adults, they appear earlier in patients with CTE [2,5].

## 3. TBI and CTE

Mechanisms regarding the potential neurodegenerative effects of acute TBI, multiple mild TBI (mTBI), or repetitive subclinical brain trauma (RSBT) are not elucidated. Proposed mechanisms include decreased cognitive reserve, chronic inflammation, chronic microglia activation, acute upregulation of amyloid precursor protein (APP) and subsequent AD-like cascades, and slow degeneration of axonal connections due to altered protein degradation processes [21]. Even in the absence of subsequent or repetitive forces transmitted to the brain, the widespread effects of TBI on neuronal homeostasis and regulatory functions suggest that one or many of these mechanisms may drive chronic dysfunction [21]. The cognitive reserve hypothesis points to the modification of the “normal aging” trajectory for the affected individual due to the effects of TBI. Additionally, multiple injuries may have synergistic effects that accelerate the rate of dementia development. TBI followed by acute neuroinflammation is associated with cytokine release, and persistent microglia activation neuropathologically, which is proven by reactive microglia found post-mortem months to years after a single TBI [22,23,24,25]. This was demonstrated by Johnson and colleagues, who identified reactive microglia in 28% of brains examined over one year following a single TBI. Similarly, others have shown that chronic inflammation and microglia activation may occur up to 17 years after TBI in areas distal to the trauma locus [23,26].

As mentioned earlier, acute TBI pathology can include Aβ and Tau deposition. The normally functioning brain has adaptive mechanisms for protein degradation and removal. For example, one of the mechanisms is the ubiquitin-proteasome pathway, an intracellular mechanism that regulates the degradation of normal and abnormal proteins, which is responsible for normal cell growth and metabolism. Due to chronic inflammation following TBI, this pathway may be impaired, resulting in an inability to efficiently clear proteins such as Aβ and p-Tau [27]. The combination of abnormal protein deposition and reduced degradation and clearance abilities suggests a possible theory that single-event moderate to severe TBI may progress to neurodegenerative processes [27].

RHI, even with mild insult, can damage axons and cause changes in membrane permeability and ionic shifts, resulting in a large influx of calcium [28]. The subsequent release of caspases and calpains is followed by events such as Tau phosphorylation, misfolding, shortening, and aggregation. Additionally, cytoskeleton failure with the dissolution of neurofilaments and microtubules may occur.

Acute head injury also activates microglia that release toxic levels of cytokines, chemokines, immune mediators, and excitotoxins such as glutamate, aspartate, and quinolinic acid. These excitotoxins inhibit phosphatases, resulting in hyperphosphorylated Tau and, eventually, neuro-tubule dysfunction and neurofibrillary tangles (NFT) deposition in various areas of the brain [29]. Hyperphosphorylated Tau abnormalities are distributed focally as perivascular NFTs and neurites at the depths of the cerebral sulci. These then spread to involve superficial layers of the adjacent cortex causing widespread degeneration in areas such as the diencephalon, medial temporal lobe structures and brainstem [30].

The glymphatic system has also been shown to be involved. Ren et al. reported that the perivascular polarization of astroglial aquaporin-4 (AQP4) is chronically disrupted in reactive astrocytes following mild and moderate TBI in mice [31]. They observed that moderate TBI caused an impaired glymphatic pathway function for >1 month after injury, which was consistent with the dependence of brain interstitial solute clearance on perivascular AQP4 [32], which slowed the clearance of interstitial solutes from the brain parenchyma [33]. This prolonged impairment of the glymphatic pathway after TBI is a key contributor to amyloid-β clearance from the brain interstitium. This process may promote early amyloid plaque deposition following severe TBI [34] and an accelerated development of amyloid pathology in an aging brain after TBI [35]. Thus, Iliff and colleagues proposed that loss of perivascular AQP4 polarization after TBI impaired the clearance of interstitial solutes along the para-vascular glymphatic pathway, including the protein Tau [33]. The inability to clear interstitial Tau, promoting intracellular Tau aggregation and neurodegeneration, further exacerbates neurocognitive decline after TBI and CTE development.

In 1969, Dr. John Olney introduced a term called excitotoxicity, which he described as a reaction that occurs in neurons after exposure to excess extracellular glutamate [36]. The excitotoxic sequalae generate high levels of reactive oxygen species and reactive nitrogen species (ROS/RNS), lipid peroxidation products (LPP), nitric oxide, and prostaglandins. These then go on to activate microglia. In addition, pathological events such as increased amyloid processing, Tau phosphorylation, microtubule disruption, membrane injury, dendritic retraction, synaptic loss, mitochondrial dysfunction, DNA injury, apoptosis, calcium dysregulation, and necrotic cell death are associated with neuronal excitotoxicity and described in TBI patients [37].

Reactive microgliosis typically results from an interaction between glutamate, cytokines and associated receptors, which can be primed by the initial traumatic head injury or other events [37]. The enhanced release of immune cytokines, chemokines, and other immune mediators, as well as a massive release of the excitotoxins—glutamate, aspartate, and quinolinic acid, follows [37]. This pro-inflammatory response accelerates neurodegeneration. Several regions, such as the frontal lobes, hippocampus, and parietal lobes, are most vulnerable to this trauma-induced immune excitotoxicity [37]. The subsequent release of ROS, RNS and LPPs interferes with glutamate clearance, resulting in a prolonged period of accelerated neurodegeneration. Repeated trauma to the brain may prevent normal microglia from switching from pro-inflammatory to reparative mode. This results in chronic microglial immune-excitotoxic activity and chronic neurodegeneration. High levels of glutamate and quinolinic acid also significantly increase the deposition of hyperphosphorylated Tau protein and contribute to the observed NFT accumulation [37].

**Figure 3:**
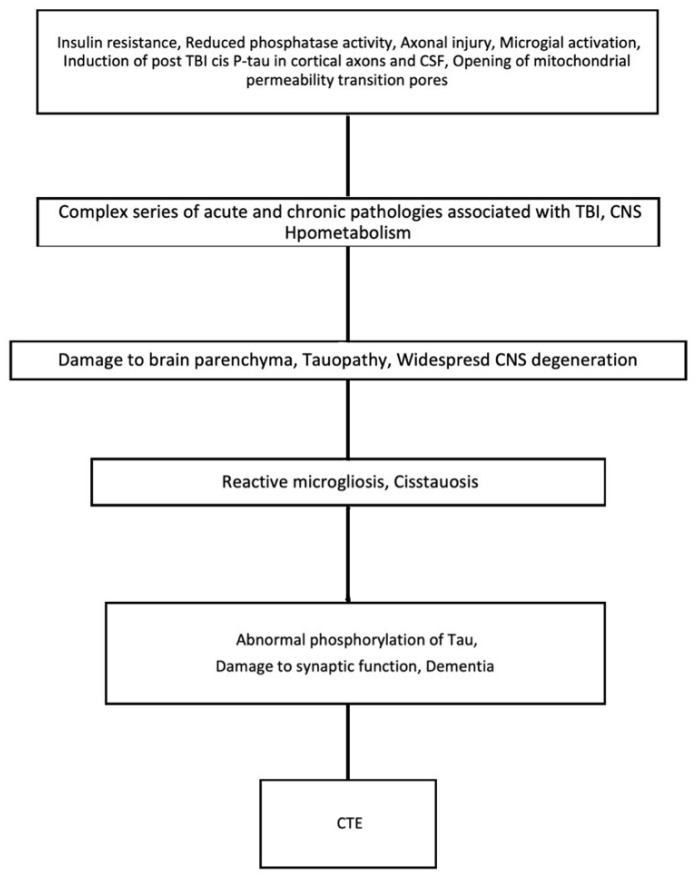
Key events which cause the progression of TBI to CTE.

Evidence of a distinctive neurodegenerative pathophysiology [38] for CTE is emerging. Within acknowledged limitations of retrospective studies thus far, it is almost exclusive to circumstances of previous exposure to TBI [39]. Despite many reports focusing on aspects of Tau-neuropathology in CTE [37], the pathology after TBI is complex. In addition to Tau, a range of abnormalities, including amyloid beta and TDP-43 deposition, neuroinflammation, axonal degeneration, white matter degradation, neuronal loss, and blood-brain barrier disruption, are deemed to be involved in the complex mechanism that is depicted below in Figure 3 [40,41,42].

## 4. Aging and CTE

The effect of aging on the development of CTE is not completely understood. The most common mechanism is cumulative change over time, with the accumulation of toxic substances, functional decline, and DNA alteration. Mitochondria have a central role in the age-related neurodegeneration and pathogenesis of CTE, and numerous studies have shown that mitochondrial changes occur with aging [43,44]. Mutations in mitochondria and associated oxidative stress contribute to the neurodegenerative process characterized by neuronal cell death, and has been described in patients with AD and PD [45].

Specifically, complex IV and V decline with age, leading to oxidative damage that can disrupt DNA and gene expression [44]. For example, changes are seen in COX gene expression that are associated with a pro-inflammatory state and neurodegeneration [46,47]. Mitochondrial mass also appears to change, as a PCR-based study reported increased content with age [48]. Furthermore, ROS accumulation leads to protein carboxylation, lipid peroxidation, and mtDNA oxidation, which are known to play a role in the development of CTE [44].

**Figure 4:**
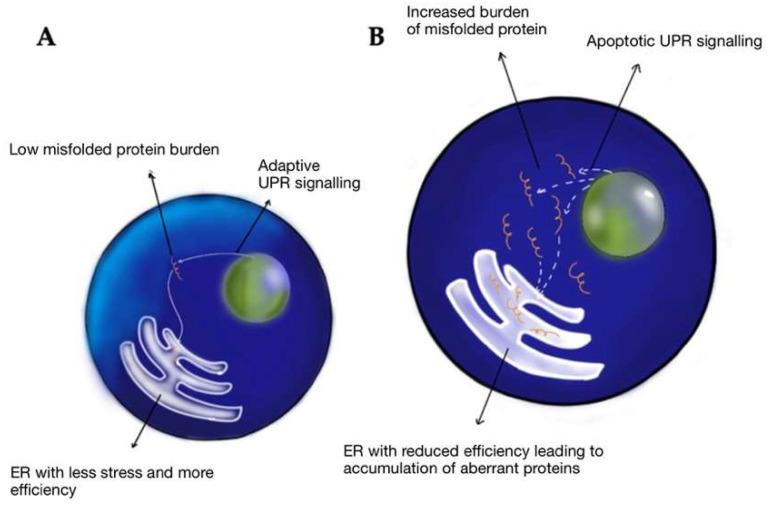
Response to misfolded proteins in the young (**A**) vs. aged (**B**) endoplasmic reticulum—when faced with misfolded proteins, young cells have low ER stress, higher chaperone efficacy, increased adaptive unfolded protein response (UPR) signaling, and increased stress tolerance. Hence, young ER is efficient in getting rid of misfolded proteins. In aged cells, due to reduced chaperone activity, the misfolded cells are unable to clear and tend to accumulate, leading to a maladaptive response as a relative increase in apoptotic UPR signaling vs. adaptive signaling, eventually leading to cell death.

The endoplasmic reticulum (ER) is a significant site of calcium storage and protein folding. Alterations in the ER environment cause stress-induced ROS production [49]. Studies have indicated that ER stress events are related to mitochondrial ROS production mechanisms within cells [49]. Ca^2+^ ions released from the ER augment the production of mitochondrial ROS to induce oxidative phosphorylation at the electron transport chain (ETC). Additionally, Ca^2+^ ions increase cytochrome c release, which impairs electron transfer, altering mitochondrial membrane potential, and increasing the generation of ROS [49]. ER stress can be provoked by TBI. This can lead to altered ER homeostasis and disrupted folding, leading to unfolded proteins and protein aggregates which are detrimental to cell survival (Figure 4) [50]. CTE is characterized by hyperphosphorylated Tau protein and, in some patients, amyloid beta-peptide. Neurons containing NFTs showed an increase in levels of free and protein-bound calcium compared with tangle-free neurons [51,52]. Calcium plays an essential role in apoptosis, neurodegeneration, and CTE.

**Figure 5:**
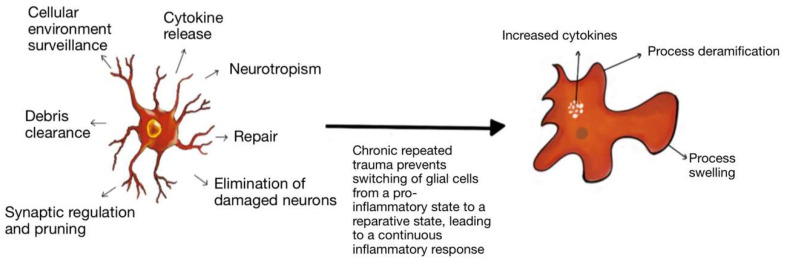
Microglial Priming: Microglia have a dual function in the brain and play a role in inflammation and repair. As microglia age, there is evidence of priming and more pro-inflammatory activity. Additionally, repeated trauma prevents the microglia from switching from its pro-inflammatory state to its reparative state, leading to a continuous inflammatory response and damage.

Microglia play a pivotal role in immune surveillance, plasticity, and development [53,54]. They have a dual function, allowing them to switch from a pro-inflammatory state to a neuroreparative state (Figure 5). In acute TBI, the microglia take part in the inflammatory process and help with the clearing of debris. Repetitive trauma does not allow the microglia to switch to their reparative mode. Hence, immunoexcitotoxicity is believed to contribute to the development of CTE [37]. There is evidence of microglia priming in the aged brain. For example, there is increased expression of inflammatory markers, including major histocompatibility complex II and complement receptor 3 (CD11), in the aging human brain [55,56]. There is also an increase in the inflammatory profile in astrocytes with age [57]. The increased inflammatory markers observed on astrocytes and microglia in the aged brain translate to an exaggerated immune response following trauma. This leads to a maladaptive response characterized by amplified and prolonged cytokine production, anorexia, prolonged social withdrawal, depressive behavior, and cognitive impairment, among other symptoms [58]. Primed microglia in an aged brain produce a more robust response to a peripheral stimulus such as stress and trauma. In addition, a study showed that in a focused laser injury mice model, there was slower migration of microglia towards the site of injury, and the microglia were aggregated at the site of damage for a longer duration than that of adult mice [59]. These functionally impaired microglia are senescent or dystrophic and indicate worse outcomes in brain injury.

Another study showed an age-dependent deficiency in the glutamate transporter on neurons of the excitatory amino acid carrier (EAAC1) [60]. It showed that genetically null mice had reduced glutathione levels and, with aging, developed brain atrophy and behavioral changes [60]. Low glutathione levels are linked to increased ROS, and ROS has been implicated in neuronal inflammation and the development of CTE. Based on our current understanding of the pathogenesis behind CTE, we can conclude that the severity of CTE is influenced by the normal aging of the human brain. Additionally, CTE is associated with the development of other neurodegenerative conditions, especially as a function of increasing age [61,62]

## 5. Neurodegenerative Diseases and CTE

Many studies have assessed the role of TBI in developing neurodegenerative conditions, and meta-analyses of these studies have shown a significant association between TBI and AD [63,64], PD, ALS [65], and FTD (Frontotemporal dementia) [66]. Pathophysiological changes of CTE have been shown to mimic molecular and cellular changes found in other neurodegenerative diseases such as AD and ALS. A study showed that microglia priming had been described in several neurodegenerative conditions, including AD. CD200 receptors are essential for switching microglia from neurodestructive mode to neuroprotective mode. Recent studies have shown reduced CD200 and CD200 microglial receptors in pathologically affected areas in AD. Macrophages of PD patients also have reduced CD200 [67].

Evidence suggests that activated microglia are a chronic source of multiple neurotoxic factors, such as tumor necrosis factor-α, NO (nitric oxide), interleukin-1β, and reactive oxygen species (ROS), driving progressive neuron damage. Microglia can become chronically activated by either a single stimulus (e.g., lipopolysaccharide or neuron damage) or multiple stimuli exposures, resulting in cumulative neuronal loss with time. This explains the prolonged activation of microglia in neurodegenerative conditions [68]. Studies have shown that pro-inflammatory immune stimulation was insufficient to cause brain pathology but triggered extensive neurodegeneration in patients with pre-existing or coexisting brain pathology in the form of excitotoxicity. Morimoto et al. found that injecting LPS plus ibotenate, an NMDA receptor agonist, led to significant neuronal degeneration and severe tissue collapse. By blocking excitotoxicity, tissue damage was prevented, despite substantial microglial activation [69]. If the ibotenate was delayed by a day after the LPS injection, gross microglial activation occurred along with significant neurodegeneration.

Tau NFT deposition is a characteristic finding identified in the brains of AD patients, as well as frontotemporal dementia (FD), Pick’s disease (PiD), and progressive supranuclear palsy (PSP), among others. These tangles have also been distinctively found in the brains of patients suffering from CTE [11]. A study done by Holf et al. discovered that the distribution of these tangles was similar to the ones found in AD and PD. However, in 2018 McKee et al. compared the brains of 68 men who had suffered a CTE to 18 age and sex-matched brains who had not suffered a CTE, discovering that brains who had undergone trauma had a unique NFT distribution that was different from any other tauopathy [2]. These included perivascular NFTs distributed in the cortex and subpial astrocytes at the sulcal depths.

Similarly, AD is also known to have AB plaque deposits around neurons, and 50% of the cases of CTE have also been found to have these deposits. However, they appear at a later disease stage and in a lesser proportion [2]. Furthermore, their presence is commonly associated with faster clinical deterioration, Lewy body formation, dementia, and parkinsonism [2].

TDP-43 is a nuclear protein that regulates the transcription of genes that bind to the E3 ubiquitin ligase Parkin mRNA to regulate its expression. The wild type of this protein has been linked to several neurogenerative diseases, such as Huntington’s disease (HD) and ALS in the hippocampus of patients suffering from AD and the limbic system in PSP (supranuclear palsy), in addition to Lewy body dementia [70]. A study done by McKee et al. studied the brains of 12 athletes aged between 42 and 85 who had developed CTE, finding that three of them had also developed motor neuron disease (MND), similar to the symptoms presented in patients who have ALS [62]. Two of the patients also developed cognitive impairment, dementia, and behavioral changes. Seven of the nine patients that did not develop MND showed TDP-43 immunoreactivity in a specific area of the brain. The athlete’s brains that developed MND all showed TDP-34 immunoreactivity throughout their brains, including the brainstem and spinal cord [62]. These findings were compared to sporadic ALS cases, in which the only difference was that none presented Tau protein tangles similar to those on CTE brains.

Moreover, it has been proven that oxidative stress plays a central role in the neuronal damage produced post-TBI. Antioxidant mechanisms are interfered with due to trauma, leading to the accumulation of ROS due to NADPH oxidase (Nox2) upregulation, which leads to DNA damage and brain inflammation [71]. In the same way, Nox2 has been proven to induce Aβ plaque formation and accumulation, which predisposes AD development. Although the final pathological manifestations of CTE closely resemble that of sporadic AD, there are some differences, especially the predominance of Tau pathology over amyloid accumulation in affected brain regions. In conclusion, CTE is a modifiable risk factor. Efforts towards developing robust biomarkers and well-designed, prospective epidemiological studies involving contact sports players from an early age to assess the risk of neurodegeneration and develop therapies are essential.

## 6. Conclusions

Chronic traumatic encephalopathy is a slowly progressive neurodegenerative condition caused by a single or multiple blows to the head. It consists of symptoms such as mood disorders, memory loss, and cognitive impairment. It is a pathological diagnosis characterized by the build-up of hyperphosphorylated Tau proteins in neuron and glial cells. Oxidative damage post-TBI plays a significant role in the development of CTE. Additionally, aging hastens the development of CTE due to changes in the endoplasmic reticulum, mitochondria, and priming of microglia, which are permissive to a pro-inflammatory response leading to repeated injury. The Tau neurofibrillary tangles found in CTE are also distinctively found in patients with neurodegenerative diseases such as AD, FTD, PiD, and PSP, among others. However, the location of these deposits varies among patients with AD and CTE.

## Data Availability

Refer to references.
